# Prenatal Nicotine Exposure Induces Low Birthweight and Hyperinsulinemia in Male Rats

**DOI:** 10.3389/fendo.2021.694336

**Published:** 2021-06-09

**Authors:** Takahiro Nemoto, Hisae Ando, Mototsugu Nagao, Yoshihiko Kakinuma, Hitoshi Sugihara

**Affiliations:** ^1^ Department of Bioregulatory Science (Physiology), Nippon Medical School, Tokyo, Japan; ^2^ Department of Endocrinology, Diabetes and Metabolism, Nippon Medical School, Tokyo, Japan

**Keywords:** nicotine, insulin, DOHaD (development origins of health and disease), sex-specificity, prenatal

## Abstract

Smoking during pregnancy is one of the causes of low birthweight. Ingestion of nicotine during pregnancy has various metabolic impacts on the fetus and offspring. According to the developmental origins of health and disease theory, low birthweight is a risk factor for developing various non-communicable diseases, including diabetes. We hypothesized that when nicotine-induced low-birthweight rats, when exposed to a high-fat diet (HFD) after growth, are predisposed to glucose intolerance as a result of a mismatch between the eutrophic environment and small body size. Therefore, we investigated whether hyperinsulinemia was caused by exposure of nicotine-induced low-birthweight rats to HFD, including whether this phenomenon exhibited possible sex differences. The average birthweight and body weight at weaning day of offspring from nicotine-administered dams was lower than those of controls. The offspring from nicotine-administered dams did not show rapid fat accumulation after exposure to HFD, and weight and body fat ratio of these animals did not differ from those of the controls. Blood glucose levels did not differ between the groups, but insulin levels increased only in male HFD-exposed offspring from nicotine-administered dams. Similarly, only in HFD-exposed male from nicotine-administered dams showed decreases in the insulin receptor expression in the liver. We conclude that male rats subjected to prenatal nicotine exposure develop hyperinsulinemia when exposed to HFD after growth. Our results suggest that decreased expression of insulin receptors in the liver may be involved in the mechanism underlying hyperinsulinemia in low-birthweight offspring, a phenomenon that appeared to exhibit a sex-specific bias.

## Introduction

There are various etiologies of low birthweight (1), and smoking by pregnant mothers is one such causes (2, 3). Smoking by pregnant mothers remains a major public health concern. Nicotine is the major psychoactive component in tobacco and believed to be the driving force for tobacco consumption. Maternal nicotine exposure is likely to have serious adverse consequences including demonstrated effect on fetal development in both humans (4) and mice (5). Fetuses exposed to nicotine are prone to some lifelong health problems, including dysfunctions in the endocrine (6, 7), respiratory (8), cardiovascular (9, 10), and nervous systems (11).

Gillman et al. have developed DOHaD (Developmental Origins of Health and Disease) into a theory intended “to recognize the broader scope of developmental cues, extending from the oocyte to the infant and beyond, and the concept that the early life environment has widespread consequences for later health” (12). At present, environmental factors at various developmental stages (including fertilization and periods of embryonic, fetal and infant development) have been shown to be associated with the health of adults and the elderly, as well as pathophysiology of various non-communicable diseases (NCDs); these effects are mediated through epigenetic alterations, and thus have come to be considered risk factors for a variety of health issues (13–15). According to the DOHaD theory, low birthweight presents an increased risk of developing various NCDs, including abnormal glucose metabolism (16). We hypothesized that exposure of nicotine-induced low-birthweight rats to a high-fat diet (HFD) after growth predisposes such animal to the development of glucose intolerance, an effect caused by a mismatch between the eutrophic environment and small body size.

Indeed, an inverse correlation has been demonstrated between birthweight and the risk of developing of type 2 diabetes (17). In addition, the association between birthweight and increased risk of type 2 diabetes is greater in low-birthweight women compared to low-birthweight men, independently of body mass index at the time of diagnosis (18). In this study, fasting blood glucose levels and hemoglobin A1c levels exhibited a strong inversely association with birthweight, with all glycemic abnormalities occurring at higher rates in low-birthweight women compared to low-birthweight men. Therefore, we also investigated whether there is a sex difference in hyperinsulinemia caused by exposure of nicotine-induced low-birthweight rats to HFD.

## Methods

### Animals

Wistar rats were maintained at 23 ± 2°C with a 12-h:12-h light-dark cycle (lights on at 0800 h, off at 2000 h). They were allowed *ad libitum* access to standard chow (rodent diet CE-2, CLEA) and sterile water. All experimental procedures were reviewed and approved by the Laboratory Animals Ethics Review Committee of Nippon Medical School (#27-067). All experiments were performed in accordance with relevant guidelines and regulations.

Female rats (9 weeks old) were anesthetized by isoflurane inhalation, and an incision was made in each animal to permit subcutaneous (s.c.) insertion of an osmotic minipumps (#2004, Alzet, Los Angeles, CA). Pumps were loaded with nicotine (Wako Pure Chemical Industries, Ltd., Kyoto, Japan), formulated in saline, at a concentration designed to deliver the drug at an initial dose rate of 3 mg nicotine/kg bodyweight/day for 28 days (starting from one week before the projected mating day through the day of delivery). Several previous papers have reported administration of nicotine to pregnant rat dams at doses ranging from 1 to 5 mg/kg bodyweight/day (19–21). For the present study, we selected a nicotine dose level of 3 mg nicotine/kg bodyweight/day per pregnant rat dams based on preliminary experiments at various dose level; notably, these data (not shown) are not detected a difference in mean food intake, and gestational age between dams maintained on vehicle compared to those maintained on nicotine at 3 mg nicotine/kg bodyweight/day. Each of control dams was implanted with an osmotic pump filled with saline and delivering the vehicle at an equivalent rate to that of the nicotine solution.

One week after pump implantation, a total of forty (20 each dosed with and without nicotine) proestrous female rats (age, 10 weeks) were mated with normal male rats. Dams were housed individually with free access to water and were divided into two groups. Twelve to twenty pups were obtained from each of 12 dams of each group. We excluded rat pups born with a body weight of more than 6.0 g, which is the mean - 2 standard deviations (SDs) body weight of the offspring of normal dams. No surrogate mothers were used, and 10 rat pups (randomly selected from a given litter) were left with each birth mother to be raised by that dam. Postnatal mother rats were fed a standard diet *ad libitum*. On the weaning day, 8 rats (randomly selected from each group) were decapitated between 09:00 am to 11:00 am and blood was collected to measure the blood GH and IGF-1 concentrations. Then, the liver was sampled to measure the expression level of IGF-1-encoding mRNA (*Igf1*). After weaning, the rat pups of different litters were mixed. Rats were divided into two groups at 5 weeks of age; one group was placed on a high-fat diet (fat-based 45kcal/fat, D12451, Research Diet), and the other on a standard diet (4.5kcal/fat, CE-2, CLEA). At 15 weeks of age, body composition was measured, blood and organs were collected, and gene and protein expression and hormone levels were analyzed. Body composition of rats was evaluated through multifrequency bioelectrical impedance analysis performed using an ImpediVet (ImpediMed, Brisbane, QLD, Australia) according to the manufacturer’s instructions (22).

### Glucose Tolerance Test

Rats were fasted for 18 hours and then subjected to glucose loading (2 g/100g bodyweight glucose) using a gastric sonde. Blood was collected from the tail vein, and the blood glucose levels before and after 15 min glucose challenge were measured (LAB Gluco glucose sensor, Filgen, Aichi, Japan).

### RNA Extraction and Real-Time Reverse Transcription-Polymerase Chain Reaction (RT-PCR)

We performed mRNA quantification as previously reported (23). Total RNA was extracted from the liver using RNAiso Plus (Takara, Shiga, Japan). The absorbance of each sample at 260 nm and 280 nm was assayed, and RNA purity was judged as the 260/280 nm ratio (The 260/280 nm ratios of all samples used in this study exceeded 1.7). First-strand cDNA was generated using 250 ;ng of denatured total RNA; the reaction mixture was incubated at 37°C for 15 min, 84°C for 5 sec, and 4°C for 5 min using a PrimeScript^®^ RT reagent kit with gDNA Eraser (Takara). PCR was performed for 40 cycles of denaturation at 94°C for 5 sec and annealing-extension at 60°C for 30 sec using SYBR premix Ex Taq (Takara) and primer sets specific for the transcripts encoding rat IGF-1 (*Igf1*; RA028844, Takara), rat insulin receptor (*Insr*; RA0.53014, Takara) and glyceraldehyde phosphate dehydrogenase (GAPDH; encoded by *Gapgh*; RA015380, Takara). To normalize each sample for RNA content, mRNA expression levels were normalized to those of *Gapdh*, a housekeeping gene. The 2^nd^ derivative method was used as the standard method for calculating C_t_ values, respectively (24).

### Western Blotting

We performed western blotting quantification as previously reported (25). Protein samples from rat liver were extracted using cOmplete lysis-M (Roche, Mannheim, Germany). The protein concentrations of lysate samples were determined using the Pierce 660 nm Protein assay (Thermo Scientific, Rockford, IL). Aliquots containing 5 µg of total protein each were electrophoresed on a 5-20% gradient SuperSep™ SDS-polyacrylamide gel (FUJIFILM Wako Pure Chemical Corporation, Osaka, Japan) and transferred to a nitrocellulose membrane. The transfer membranes were blocked with 5% skim milk and then incubated with an anti-insulin receptor (Insr) antibody (1:1,000, Abcam, Cambridge, UK, Cat# ab131238, RRID: AB_11155955) for 1 h at room temperature. The transfer membranes were washed with Tris-Buffered Saline with 0.05% Tween 20 (TBS-T, pH7.6) and further incubated with horseradish peroxidase (HRP)-labeled anti-rabbit IgG (1:5,000, Jackson ImmunoResearch Labs, West Grove, PA, Cat#711035152, RRID: AB_10015282) for 1 h at room temperature. The signals were detected using SuperSignal West Dura extended duration substrate (Thermo Scientific). Following detection, the membranes were stripped of the antibody using Restore PLUS Western Blot Stripping Buffer (Thermo Scientific). The stripped blots then were re-probed using THE™ [HRP]-labeled anti-β-actin antibody (1:1,000, GeneScript, Piscataway, NJ, Cat# A00730-40),. The expression levels of Insr were normalized to those of the β-actin signal.

### Measurement of Blood GH, IGF-1 and Insulin Levels

Hormone levels were measured using serum derived from blood obtained from decapitated rats. Serum GH and IGF-1 were analyzed using a Rat GH ELISA kit (Shibayagi, Co, Ltd., Gunma, Japan) and a Mouse/Rat IGF-1 Immunoassay kit (R&D Systems, Minneapolis, MN), respectively. Insulin was measured using a rat insulin EIA kit (Macrodia AB, Uppsala, Sweden).

### Statistical Analysis

Unpaired *t* tests, a one-way analysis of variance (ANOVA) followed by Tukey’s *post hoc* test for multiple comparisons, or ANOVA were used for each statistical analysis. All analyses were performed as two-tailed test. Prism 9 software (GraphPad Software, Inc., La Jolla, CA) was used for all calculations. Real-time RT-PCR and western blot data are expressed as percent ± SEM with the control defined as 100%. p <0.05 was considered statistically significant.

## Results

**Figure 1:**
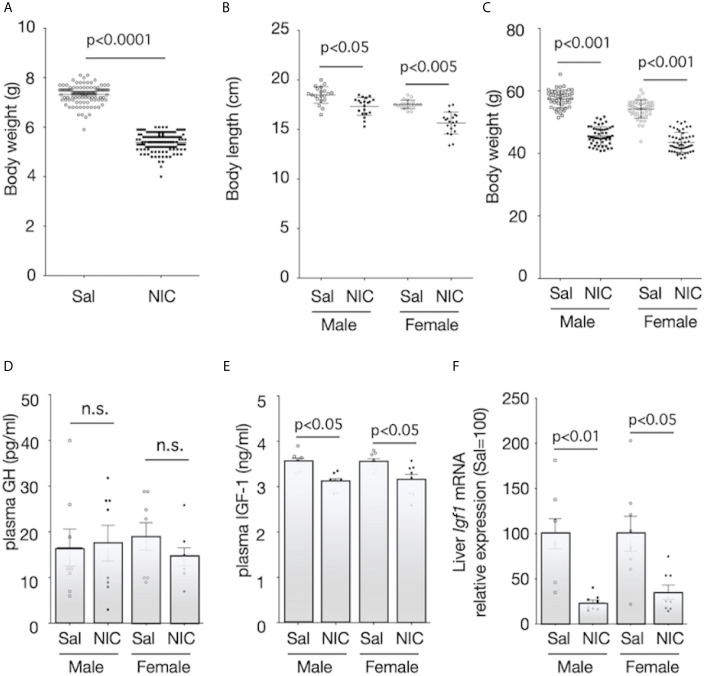
Body size and plasma concentrations of GH and IGF-1, and expression of IGF-1 mRNA in the liver. Bodyweight at birthday **(A)**, body length **(B)** and bodyweight at weaning day (3-week-old) **(C)** were measured. Plasma concentrations of growth hormone (GH) **(D)** and insulin-like growth factor (IGF)-1 **(E)** of offspring from nicotine-administered dams (NIC) and saline-treated dams (Sal) were measured. The mRNA expression levels of *Igf1* in the liver of offspring from nicotine-administered dams (NIC) and saline-treated dams (Sal) were quantified **(F)**. The mRNA expression levels were normalized to *Gapdh* levels and then to that in Sal, which was defined as 100%. Values are presented as means ± SEM (n=8). Statistical analysis was performed using unpaired-T test **(A)** and one-way ANOVA followed by Tukey’s *post hoc* test for multiple comparisons **(B–F)**. n.s., not significant (p ≥ 0.05).

During pregnancy, there were no statistically significant differences in food intake and during pregnancy between the nicotine-administered (7.0 ± 1.1g/100g b.w.; mean ± SEM, n=10) and saline-treated groups (7.7 ± 0.6g/100g b.w., p≥0.05, n=10). Similarly, changes in bodyweight during pregnancy were not significantly different between nicotine-administered dams and control dams (p>0.05, n=10 of each dams). Litter size was equivalent between groups. The mean bodyweights of the pups on the day of birth were 5.38 ± 0.03 g (n=118) and 7.33 ± 0.04 g (n=108) for offspring of the nicotine-administered dams and the control dams, respectively (**Figure 1A**). The body length and the bodyweight at weaning day (at 21 days of age) of male rats were significantly lower in offspring from nicotine-administered dams than in offspring from control dams (**Figures 1B, C**). Similarly, the body length and the bodyweight at weaning day of female rats were significantly lower in offspring from nicotine-administered dams than in offspring from control dams (**Figures 1B, C**). The pups’ serum GH levels at the weaning day did not differ significantly between the groups (**Figure 1D**), but serum IGF-1 levels (**Figure 1E**) and the expression levels of *Igf1* mRNA in the liver (**Figure 1F**) were significantly lower in both male and female offspring from nicotine-administered dams than in offspring from control dams.

**Figure 2:**
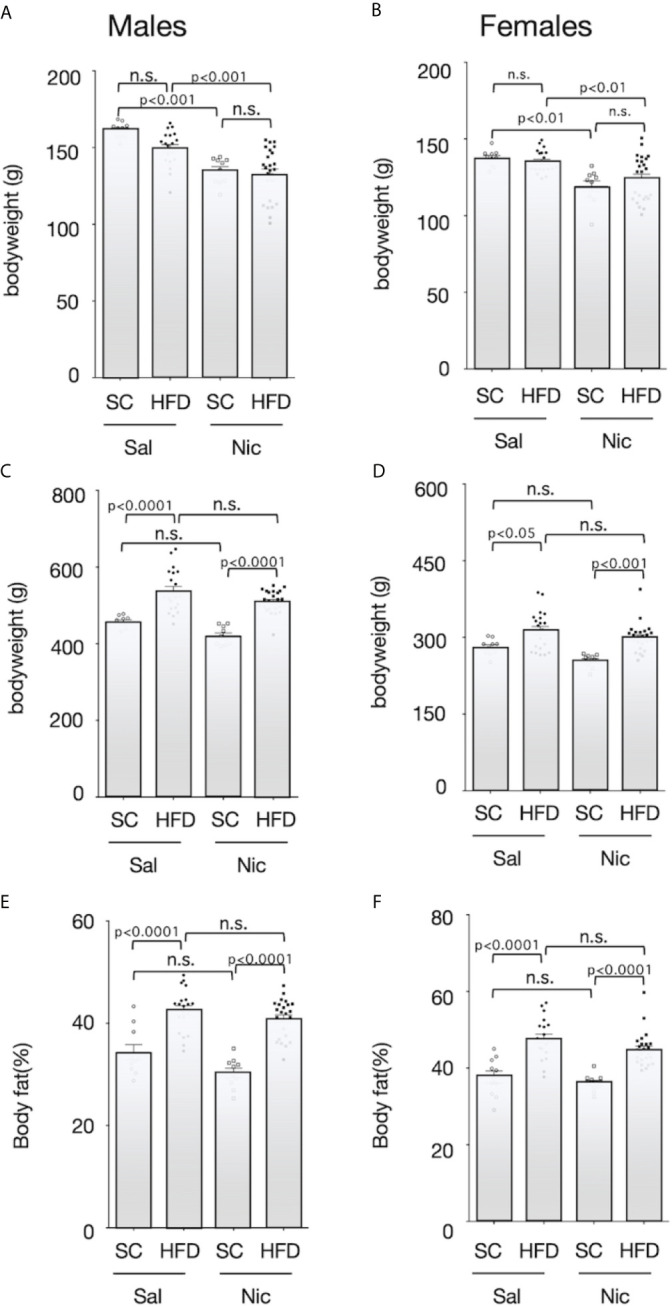
Bodyweights and body fat ratio. Plots of the bodyweight before (5-week-old) (**A** for males and **B** for females****) and after (13-week-old) (**C** for males and **D** for females****) exposure to the high-fat diet (HFD) or standard chow (SC), and the body fat percentage after exposure to the HFD or SC (13-week-old) (**E** for males and **F** for females****) are shown. Values are presented as means ± SEM. Statistical analysis was performed using one-way ANOVA followed by Tukey’s *post hoc* test for multiple comparisons. n.s., not significant (p ≥ 0.05).

At 5 weeks of age, the pups were randomly divided into two groups; and maintained on either HFD or standard diet. Body weight before HFD exposure was significantly lower in offspring from nicotine-administered dams than that in offspring from control dams (**Figures 2A, B**). HFD significantly increased the bodyweight of the offspring from both the nicotine-administered dams and the control dams, but there was no significant difference between the offspring from the nicotine-administered dams and those from control dams (**Figures 2C, D**). In addition, HFD significantly increased the body fat ratio in both males and females, but there was no significant difference in the magnitude of these increase between offspring from the nicotine-administered dams and those from control dams (**Figures 2E, F**).

**Figure 3:**
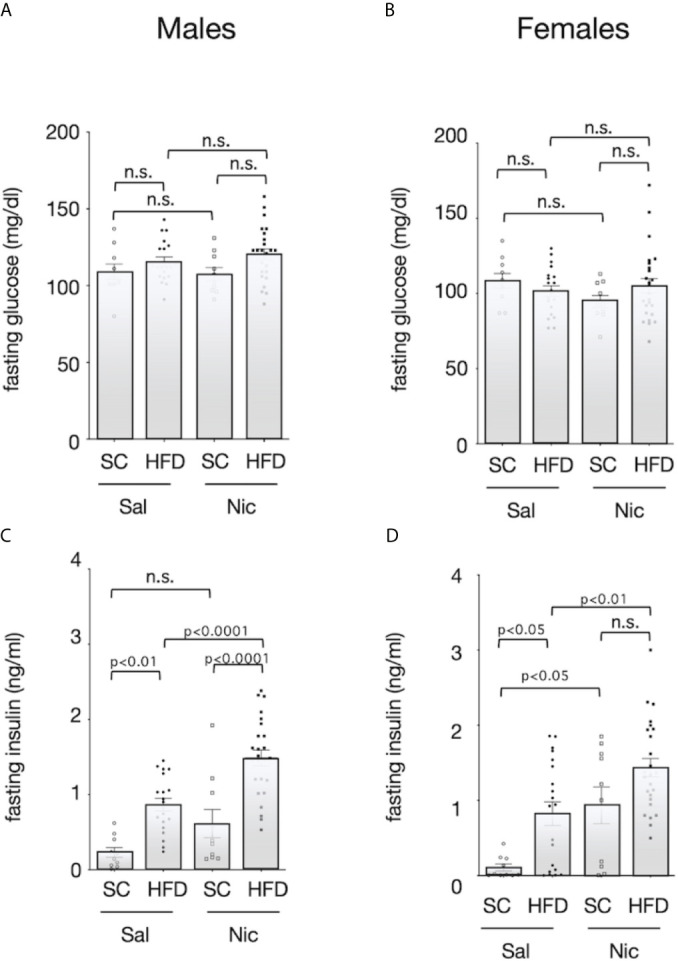
Fasting blood sugar and insulin levels. Plasma concentrations of glucose (**A** for males and **B** for females****) and insulin (**C** for males and **D** for females****) of standard chow-fed (SC) offspring or high fat diet (HFD)-exposed offspring from nicotine-administered dams (NIC) or saline-treated dams (Sal) were measured (13-week-old). Values are presented as means ± SEM. Statistical analysis was performed using one-way ANOVA followed by Tukey’s *post hoc* test for multiple comparisons. n.s., not significant (p ≥ 0.05).

There was no significant difference in fasting blood glucose among the groups (**Figures 3A, B**). Fasting blood insulin levels were significantly higher in HFD-exposed male offspring of both the nicotine-administered dams and of control dams than in standard chow-fed offspring of each set of dams. In contrast, there was no significant difference in blood insulin levels between the standard diet-fed offspring of nicotine-administered dams and of control dams (**Figure 3C**). Among females, fasting insulin levels were higher in offspring from the nicotine-administered dams than in offspring of controls dams. Among the offspring of control dams, fasting insulin levels were significantly higher in the HFD-exposed rats than in standard chow-fed rats; in contrast, no significant diet-associated difference was observed in offspring of nicotine-administered dams (**Figure 3D**).

**Figure 4:**
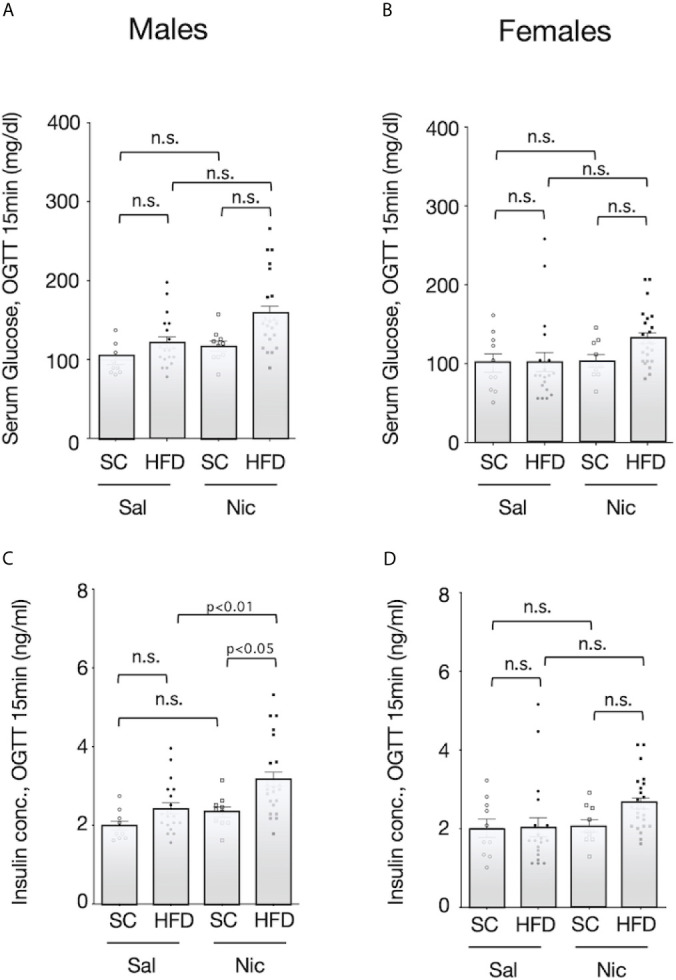
Blood sugar and insulin levels after oral glucose challenge. Blood samples were collected 15 min after glucose solution (2g/100g b.w.) ingestion. Their plasma concentrations of glucose (**A** for males and **B** for females****) and insulin (**C** for males and **D** for females****) of standard chow-fed (SC) offspring or high fat diet (HFD)-exposed offspring from nicotine-administered dams (NIC) or saline-treated dams (Sal) were measured. Values are presented as means ± SEM. Statistical analysis was performed using one-way ANOVA followed by Tukey’s *post hoc* test for multiple comparisons. n.s., not significant (p ≥0.05).

Oral glucose challenge (2g/100g b.w.) did not reveal significant differences between HFD exposed rats and standard chow-fed rats or between offspring of nicotine-administered and control dams when blood glucose levels were assessed 15 minutes post-challenge (**Figures 4A, B**). Among male HFD-exposed rats, blood insulin levels in response to glucose challenge were significantly higher in offspring of nicotine-administered dams than in offspring of control dams. Moreover, among offspring from nicotine-administered dams, the blood insulin concentration in response to glucose challenge was significantly higher in HFD-exposed rats than in standard chow-fed rats (**Figure 4C**). However, there were no significant differences in insulin levels in female rats when comparing between the groups based on dams or based on diet (**Figure 4D**).

**Figure 5:**
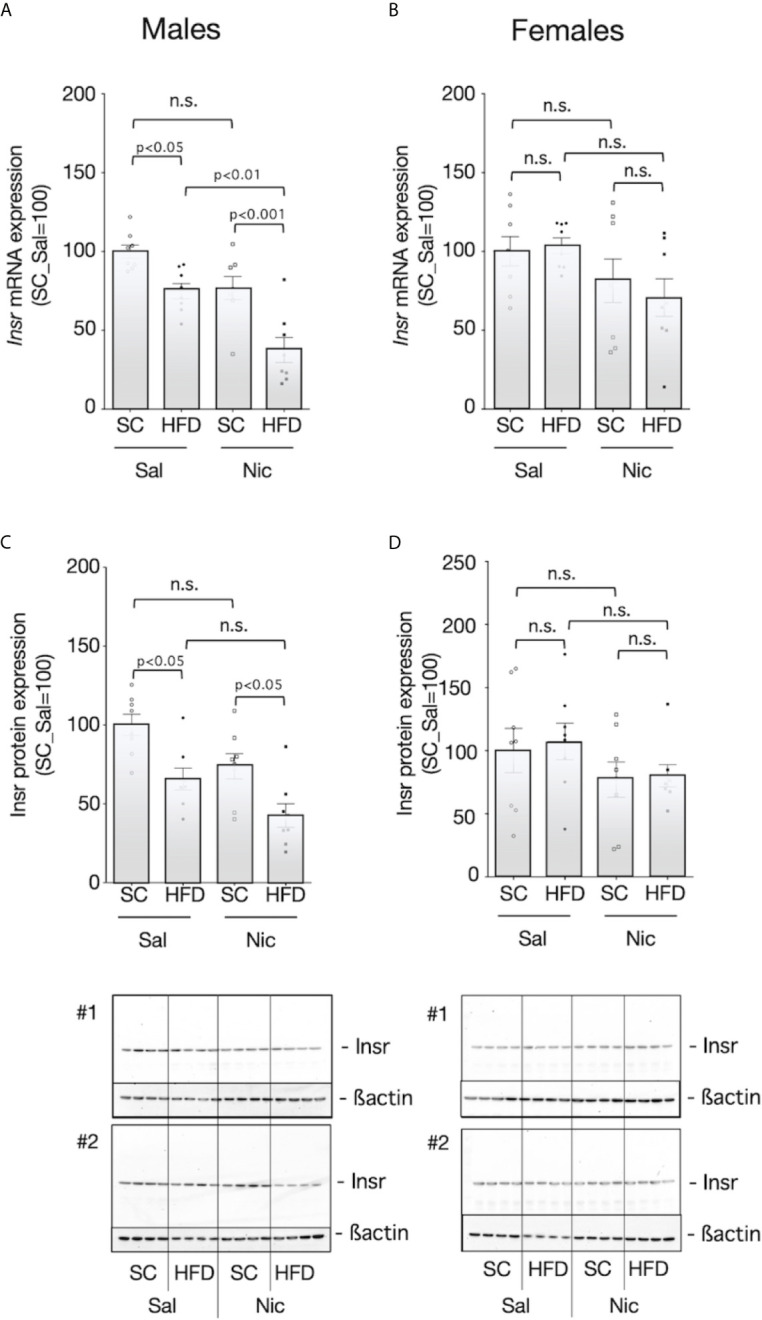
Expression of insulin receptor (Insr) in the liver. *Insr* mRNA expression levels (**A** for males and **B** for females****) and Insr protein expression levels (**C** for males and **D** for females****) in the liver of standard chow-fed (SC) offspring or high fat diet (HFD)-exposed offspring from nicotine-administered dams (NIC) or saline-treated dams (Sal) were quantified. The mRNA expression levels were normalized to Gapdh levels and then to that in Sal, which was defined as 100%. The protein expression levels were normalized to that of ß-actin and then to that in Sal, which was defined as 100%. Values are presented as means ± SEM (n=8). Statistical analysis was performed using one-way ANOVA followed by Tukey’s *post hoc* test for multiple comparisons. n.s., not significant (p ≥ 0.05).

The liver expressions levels of the *Insr* mRNA and Insr protein were significantly lower in HFD-exposed offspring than in standard chow-fed offspring from nicotine-administered dams and control dams (**Figures 5A, C**). As seen with blood insulin levels, there was no significant difference in the *Insr* mRNA and Insr protein expression in female rats when comparing between the groups based on dams or based on diet (**Figures 5B, D**).

## Discussion

**Figure 6:**
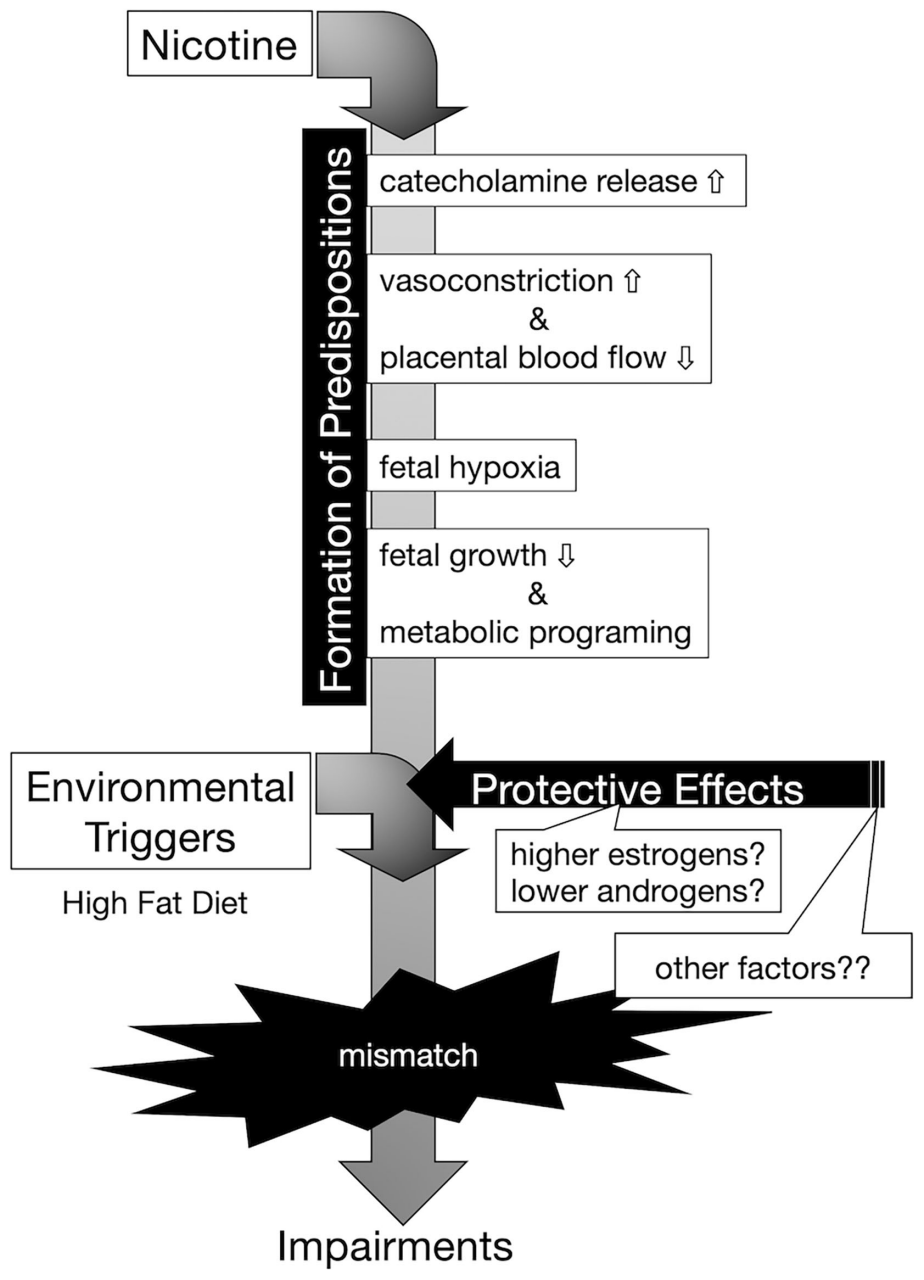
Conceptual diagram of the onset of hyperinsulinemia due to embryonic nicotine exposure. Exposure to nicotine in dams causes a decrease in uterine blood flow, and metabolic programming due to hypoxia and malnutrition in the fetus is assumed. Nicotine may directly affect the egg *via* cord blood, but the effect is unknown in this study. According to DOHaD theory, the mismatch between the predispositions acquired by low-birthweight offspring and the post-growth environment increases the risk of developing the disease. Female hormones (estrogens) may act protectively in the development of diabetes. In our experimental model, it is possible that the decrease in the expression of insulin receptors in the liver was blocked in the female rats compared to that of male rats, but since there was no difference in the DNA methylation of the insulin receptor gene, further analyses are needed to clarify the mechanism underlying the sex-specificity in the future.

In this study, offspring from nicotine-administered dams delivered low-birthweight offspring, and those offspring failed to “catch up” in growth by the weaning day. Among the pups, there was no significant difference in GH at the weaning day, and the levels of Igf1 mRNA on the liver and of IGF-1 in serum were lower in low-birthweight rats delivered from nicotine-administered dams (compared to those in pups delivered from control dams). It has been reported that, in the offspring of dams in which the uterine arteries were ligated in the last trimester, blood IGF-1 levels decrease during weaning; epigenetic changes in the *Igf1* promoter region are thought to be involved in the decrease in IGF-1 production (26). Although we did not analyze epigenetic changes in the *Igf1* gene as part of the model used here, the changes in the epigenome may have reduced *Igf1* mRNA expression levels. In fact, Ernst, et al. reviewed possible evidence that nicotine activates maternal peripheral nicotinic acetylcholine receptors, resulting in increased catecholamine release, followed by vasoconstriction and decreased placental blood flow, causing fetal hypoxia, which can further impair fetal growth (**Figure 6**) (27). These authors explained that nicotine tends to disrupt the function of the placenta. The activation of placental cholinergic systems by nicotine is known to suppresses transplacental amino acid transport. This reduction of placental amino acid transport may contribute to fetal intrauterine growth retardation. Thus, we postulate that the decreases in umbilical cord blood flow and nutrient transport due to nicotine exposure in dams decreases IGF-1 production in offspring and may impaired catch-up growth.

In the present study, we showed that exposure to a HFD of male offspring from nicotine-administered dams significantly increased blood insulin levels in response to glucose challenge without associated rapid accumulation of body fat. However, we observed that low-birthweight female offspring from nicotine-administered dams did not exhibit increased blood insulin levels after oral glucose challenge, even when exposed to an HFD. The expression level of insulin receptor in the liver also was decreased in HFD-exposed male offspring from nicotine-administered dams, but not in HFD-exposed female rats. These results suggested that there are sex differences in the effects of nicotine exposure on fetal programming. Namely, in our prenatal nicotine-exposed rat model, males may have a higher predisposition to develop insulin resistance than females. Indeed, in humans, type 2 diabetes develops slightly more frequently in males, and males develop the disease at a younger age than do females (28, 29). On the other hand, female-specific changes in the pathophysiological mechanism of human diabetes also have been clarified. A large genome-wide association studies (GWAS) meta-analysis in individuals of European descent identified genetically coded sexual dimorphisms for metabolic characteristics and outcomes, including female-specific effects on type 2 diabetes (30). It is becoming clear that not only are there sex differences in the onset of the disease, but also that the onset of the disease in the offspring is affected by the uterine environment; sex differences in dysregulation of gene expression have been demonstrated in numerous animal models. Both male and female offspring from type 1 diabetes dams showed reduced insulin secretion responded to oral glucose. However, in that model, only female rats (but not males) showed decreased insulin secretion in response to intravenous glucose infusion (31). Another model demonstrated that HFD combined with maternal vitamin D deficiency disrupted glucose homeostasis and adiposity in male offspring but not in females. The authors of that work showed that such phenotypes were associated with different transcriptomic profiles in adipose tissue, which could be related to differential modulation of plasma 17ß-estradiol concentrations (32). In fact, insulin sensitivity and beta cell function are known to be higher in females than in males (33). Mechanistically, estrogens have been reported to protect against insulin resistance through activation of the estrogen receptor (ER) α pathway in insulin-sensitive tissues (34, 35). Also, ERα signaling in hepatocytes mediates protective effects against steatosis and insulin resistance in HFD-exposed female mice (36). On the other hand, it has been reported that growth-restricted in males are resistant to impaired glucose homeostasis, whereas growth-restricted females are susceptible to metabolic dysfunction regardless of postnatal diet (37). The difference in these results suggests that the cause of growth retardation may be malnutrition or other factors. In the present work, we observed that low-birthweight male rats (but not females) resulting from embryonic nicotine exposure had elevated blood insulin levels when exposed to an HFD. In this study, chronic neonatal nicotine exposure did not produce sex-specific differences in changes of body weight, or overall growth pattern. Therefore, we postulate that there was a difference in predisposition to glucose intolerance after birth attributable to the protective effect of estrogen, and not as a result of the effect of the decrease in GH and/or IGF-1 levels due to fetal programming by nicotine (**Figure 6**). Further studies using ovariectomized rats or aged female rats are needed to investigate the protective effect of estrogen on glucose tolerance in nicotine-exposed low-birthweight rats.

In the present study, there were no significant differences in fasting blood glucose, nor in mRNA and protein expression levels of the insulin receptor in the liver, when comparing between the standard diet-fed low-birthweight rats and the standard diet-fed control rats. The concept of the DOHaD suggests that adverse influences early in development, particularly during the intrauterine stage, may result in changes in the physiological and metabolic responses of the fetus, which in turn may result in an increased risk of NCDs after growth. Understanding of the underlying molecular mechanisms of fetal malnutrition and the impact of low birthweight on the development of NCDs in later years is incomplete. Organisms have an evolved ability to respond to environmental changes by adjusting their phenotype during development. Therefore, a fetus exposed to a signal that the fetus interprets as reflecting undernourishment or maternal chemical exposure is expected to result in an adaptation of the prenatal metabolic systems to suit the environment. Subsequent maladaptation occurs when the postnatal environment does not match the prenatal environment, leading to obesity and type 2 diabetes (38). In humans, the combination of low birthweight and rapid growth in childhood is associated with later insulin resistance (39). In this study, there were no statistical differences between fasting and blood glucose levels after the oral glucose challenge. This means that glucose tolerance remains normal although insulin sensitivity is reduced in the offspring from nicotine-administered dams. Furthermore, the initial secretion of insulin after the oral glucose challenge was not reduced in high-fat diet exposed offspring from nicotine-administered dams, indicating that pancreatic β-cells were maintained normally even when the fetus is exposed to the nicotine. Thus, the DOHaD theory suggests that the onset of NCDs is triggered by a two-step process involving a combination of the embryonic environment and the postnatal environment, and that its onset takes time. The results of the present study indicate that impaired glucose tolerance due to fetal nicotine exposure manifests as a result of a second hit of HFD exposure. In other words, embryonic programming by nicotine exposure may affect the gene expression regulation mechanism. The expression level of the insulin receptor in the livers of our embryonic nicotine-exposed rats was decreased in HFD-exposed rats. The induction of altered phenotypes during development in response to environmental stimuli is accompanied by epigenetic changes. Epigenetic factors include DNA methylation and histone modifications. Epigenetic changes, especially DNA methylation, provide a “memory” of developmentally plastic responses to the early environment and are central to phenotypic generation and their stability throughout the life course (40, 41). Animal studies have reported that changes in gene promoter methylation induced in the F1 generation by maternal protein restriction during pregnancy are transmitted to the F2 generation. This result represents a mechanism of phenotypic induction of transmitted between generations (42). In humans, it has been reported that increased DNA methylation is involved in decreased insulin receptor expression in the adipose tissue of infants born to mother with gestational diabetes (43). However, the degree of change in DNA methylation is very small. In fact, we also quantified DNA methylation (using bisulfate sequencing) in the promoter region of *Insr* in rat liver, but the observed differences contributed only a few percent of the eligible nucleotides (data not shown in figures). Therefore, it is possible that something other than DNA methylation (i.e., methylation of histone H3 K27, which is known to affect transcription elongation) may be involved. Clearly, further research will be needed to address the mechanism of the effect observed in the present study.

As shown in this study, embryonic nicotine-exposed rats exhibit hyperinsulinemia when exposed to an HFD after growth. Our results suggest that decreased expression of insulin receptors in the liver may be involved in the formation of predisposition of insulin resistance; notably, the observed effects revealed a sex-specific difference in the developmental outcomes among fetal nicotine-exposed low-birthweight offspring. Hence, moderation of fat and sugar intake may be warranted in those, especially males, born with low birthweight, especially for males, to ensure minimal risk for type 2 diabetes.

## Data Availability Statement

The raw data supporting the conclusions of this article will be made available by the authors, without undue reservation.

## Ethics Statement

The animal study was reviewed and approved by the Laboratory Animals Ethics Review Committee of Nippon Medical School.

## Author Contributions 

TN designed the work, acquired data, analyzed data, and drafted the manuscript. HA performed western blot analysis of the liver. MN designed the work and drafted the manuscript. YK and HS interpreted the data and substantively revised the manuscript. All authors contributed to the article and approved the submitted version.

## Funding

This work was supported in part by the Smoking Research Foundation of Japan (grant ID: 2014G003).

## Conflict of Interest

The authors declare that the research was conducted in the absence of any commercial or financial relationships that could be construed as a potential conflict of interest.
